# Tuberculosis of Joints

**Published:** 1907-05

**Authors:** 


### TUBERCULOSIS OF JOINTS.

T. E. Potter, in the Medical Herald, considers this vexed subject. It was formerly taught that wherever there was tubercular arthritis, there was in the person, strictly, a scrofulous diathesis. An extensive search was always made to find a history of some of the patient's ancestry, who had had tuberculosis, or this constitutional taint—scrofula; but we now know that the healthiest man or woman cannot always resist tubercular infection, and if exposed to the germs, may, in some manner, become their slave. And, if once infected by them in any way, they may show themselves in some organ or part that does not have the proper amount of resisting force. So a healthy individual, if inoculated, may have a disabled joint from injury, or disease, or said joint may be weakened from an inherited taint, and the patient may find it difficult to give a satisfactory history why he should be affected with tubercle.

Two boys may be playing and both have a knee or ankle joint slightly injured. One gets well without being seriously discommoded, and the other becomes afflicted with a chronic tubercular arthritis. In this case the question presents itelf at once—Was he constitutionally affected, or was it from the local injury? If it were from the local injury we all know the tissues would repair themselves, and the parts would soon be well, unless on receiving the injury, there was inoculation. Then there must be in the blood tubercular germs or spores waiting a condition for favorable development.

How long these germs will be permitted to remain in the tissues without in some way manifesting themselves, is one of those mysteries that have not been solved by science; and, as to the length of time which these germs or spores may live without attacking tissues some way or some how, cannot be well understood. Another unsolved question is, How long will they live under proper conditions without losing their power to reproduce themselves? Years have been known to intervene between the time when we were satisfied parties were infected, before there was any decided manifestation of the trouble. Leneck in making a post mortem examination upon a tubercular subject allowed the saw to wound his finger, and from this wound a tubercle grew and was destroyed. He lived afterwards for twenty years, when he died of pulmonary tuberculosis. We have known children to give evidence of tuberculosis by having enlarged tubercular glands, get apparently well, and in years afterwards die of tubercular consumption, or have tubercular osteomyelitis or synovitis. A Frenchman has recently excited the scientific world in his own country by declaring that the "white plague" which we are all so much interested in trying to control was not known until the imperial tombs of Egypt were invaded by relic hunter and scientist, and from the dust of mummies, this disorder had spread over the entire civilized world. He shows that tuberculosis was not known in France until Napoleon's army brought these antiquated bodies to that country, and instead of being harmless, he claimed they spread the contagion throughout that entire nation and then to other countries.

If there is truth in this statement, it would seem that life in these germs under favorable conditions may live an indefinite period. We, therefore, conclude that it is reasonable to suppose that infection may take place, first, by predisposition; second, by food and drink; third, by inhalation; fourth, by inoculation.

With these methods of infection who can say that he himself may not be the custodian of these germs? All slight injuries to the joints should be particularly watched, for they are much more conducive to tubercular arthritis than extensive ones. In the former, the tissues are only weakened, and for repair no great amount of activity takes place, while in the latter more blood is invited to the damaged structure and the activity in the way of repair is greater, and germ life is more readily destroyed by the great number of leucocytes drawn to such parts for this purpose. So we conclude that tuberculosis of joints takes place by the germs, or spores, being carried to them by the blood introduced by one of the four ways mentioned above.

It does not follow in this assault upon a joint that they by any means begin at first to damage the synovial membrane. This comes afterwards. They find a convenient place to do their work by depositing themselves in the cancellous tissue, and in Howship's lacuna, and here multiply most rapidly, consuming the nourishment and shutting off the blood supply in the spaces and cells intended for free and undisturbed nutrition and circulation in these bony parts.

Then we soon see the proximal tissues, osseous, muscular, and membraneous taking on the conditions as described by Wiseman, and quoted in the beginning of this paper. A convenient and common-place site in children for these germs to assert themselves is between the epiphysis and diaphysis, and they may get in their work so well as to completely arrest the growth of the bone in length by separating the epiphysis from the diaphysis and by destroying the former.

The symptoms of tuberculosis of the joints may be mild, and cause the practitioner at first to suspect rheumatism; but the fact that the trouble is only confined to one joint should awaken suspicion in his mind. Further, at the first visit we are very likely to think the suffering and pain is caused by a slight sprain from jumping, or some trivial accident; but we should not be too ready to dismiss the matter with this statement. The appearance of the child, and family history will help along this line, but not always. If the child is very precocious, or if there is in the family a history of tuberculosis, the doctor should move along very cautiously in his study of the case and not give too favorable a prognosis. If the trouble still lingers after keeping the patient quiet for a day or two, and at the same time using local applications as heat and remedies to allay pain, then grave suspicions as to it being tubercular should enter the mind. The drawing of the tendons, the enlargement of the bone next to the joint, and the continuous swelling and pain in and around the joint are strong symptoms. Hydrops which may take place sooner or later is evidence of the true cause. Fungus growths in the joints or floating cartilages that may and are often felt, either with or without hydrops, are valuable symptoms to confirm diagnosis. Pus may show itself if there is mixed infection, which is noticed in many cases.

As to the prognosis, some cases, no doubt, recover spontaneously; others with but slight improvement leaving the patient with partial ankylosis or contraction of the tendons, as well as enlargement of joint, or a few floating cartilages within the synovial sack. These occasionally give trouble, but may prove to be most serious and extensive, and require constant attention and radical treatment.—Med. Standard, April, 1907.

